# Direct to psychology stepped care model implementation in the multidisciplinary sleep service: study protocol

**DOI:** 10.1186/s12913-025-13134-5

**Published:** 2025-08-18

**Authors:** Sara Winter, Sara Crocker, Tricia Rolls, Deanne Curtin, Jessica Haratsis, Tamlyn Rautenberg, Irene Szollosi

**Affiliations:** 1 Allied Health Research Collaborative, The Prince Charles Hospital, Brisbane, Australia; 2 Psychology Department, The Prince Charles Hospital, Brisbane, Australia; 3 Sleep Disorders Centre, The Prince Charles Hospital, Brisbane, Australia; 4 Metro South Addiction and Mental Health Services, Brisbane, Australia; 5 School of Biomedical Sciences, University of Queensland, Brisbane, Australia; 6 School of Medicine and Dentistry, Centre for Applied Health Economics, Griffith University, Brisbane, Australia; 7 Faculty of Medicine, University of Queensland, Brisbane, Australia

**Keywords:** Sleep disorders, Sleep psychology, CBTi, Stepped care, Service redesign, Insomnia, Implementation science

## Abstract

**Background:**

Sleep disorders are common and costly to society. Guidelines from the American Academy of Sleep Medicine (AASM) and the Australasian Sleep Association (ASA) recommend Cognitive Behaviour Therapy for Insomnia (CBTi) as the best treatment modality for insomnia disorder in the long term, however, access issues due to medical first referral pathways and psychology workforce shortages in the community result in increased wait times, reduces efficient care for patients, and adds burden to specialist medical services.

**Method:**

This single-site, multiphase, mixed methods study will implement and evaluate a service model of care re-design incorporating a new Direct to Psychology referral pathway for insomnia presentations and a Stepped Care treatment approach including digital intervention in a quaternary public multidisciplinary sleep service. The RE-AIM framework and Standards for intervention Reporting Implementation Studies (StaRI) will be used to guide the development, implementation, evaluation and reporting of this service change. Quantitative data measuring outcomes of the new models of care on key indices of patient clinical outcomes, patient/clinician service satisfaction, and service efficiencies will be collected pre- and post-service change.

**Discussion:**

The overarching aim of this project is implement and evaluate a model of care change to the service model in a public tertiary hospital multidisciplinary sleep service. The new Direct to Psychology Stepped Care Model incorporates a Direct to Psychology Insomnia referral pathway along with a Stepped Care approach to treatment, flexibly delivered across the continuum of care. The project will measure the impact of the service change in our target population. The new model of care will be compared to the old service model for key outcomes. We anticipate that the new Model of Care will be acceptable to stakeholders, will produce non-inferior patient outcomes and experience, and will improve patient flow and service efficiency.

**Trial sponsor:**

Psychology Department, The Prince Charles Hospital, Rode Rd, Chermside, QLD, Australia, 4032. The sponsor had no role in the study design; data collection, management, analysis, or interpretation; writing of this or future reports; the decision to submit for publication; or ultimate authority over any of these activities.

**Trial registration:**

The Protocol was registered on 5/08/2022 with the Australian and New Zealand Clinical Trials Registry (ACTRN12622001086752). The study was conducted in accordance with the protocol version 4: 12 March 2024.

**Supplementary Information:**

The online version contains supplementary material available at 10.1186/s12913-025-13134-5.

## Background

Insomnia is one of the most common sleep disorders globally [1, 2], with up to 15% of the population diagnosed every year. The total health system cost in Australia due to sleep disorders was estimated to be $0.9 billion in 2019-20, 30% of which was attributable to insomnia [3]. Insomnia is a direct predictor of the development and exacerbation of mental health disorders [4], and psychological treatment of insomnia is associated with improved mental health outcomes and prevention of relapse in the case of comorbid depression [5] and anxiety, even if the depression or anxiety itself is not targeted for intervention [5, 6]. In the case of comorbid insomnia and Obstructive Sleep Apnoea (OSA) (COMISA), psychological intervention for insomnia is associated with significantly increased acceptance and adherence to Continuous Positive Airway Pressure therapy (CPAP), the gold standard treatment for OSA [7].

Sleep Psychology is a necessary resource within any sleep disorders service, with AASM guidelines recommending a Psychologist with experience in behavioural sleep medicine be embedded in multidisciplinary sleep services [8]. However, barriers to patient access to treatment include limited availability of appropriately trained and experienced psychologists [9] and lack of knowledge amongst primary practitioners about evidence-based treatment for insomnia [10]. To this end, it is important to explore options for improving efficiencies and access to care for individuals requiring intervention.

There is significant evidence for Cognitive Behaviour Therapy for insomnia (CBTi) as the first-line treatment for insomnia [2, 11], recommended by both the American Academy of Sleep Medicine (AASM) and the Australasian Sleep Association (ASA), as CBTi has extensive Level I evidence for longitudinal gains in insomnia resolution [8, 12]. Internet-delivered CBTi has been extensively investigated in the clinical literature, with systematic reviews and meta-analyses demonstrating that these first-line interventions are just as effective as face-to-face modalities in the general population for those with insomnia of low to moderate severity [6, 13–18]. “Stepped care” refers to a service delivery model allowing a relatively scarce healthcare resource to be applied in a cost-effective way to achieve timely and effective clinical care [15, 19]. CBTi is a well validated intervention with demonstrated amenability to a Stepped Care model, where patients are assessed for entry to first-line manualised and online treatment programs, increasing to progressively more time, cost and expertise intensive levels ‘upstream’ where there is an incomplete therapeutic response to a first-line intervention, or when clinical complexity demands a more intensive intervention step within the care pathway.

In our tertiary public hospital multidisciplinary sleep service, the current model of care (MoC) relies on a “medical-first” referral pathway which overburdens specialist medical services and delays care to evidence-based psychological treatment. Furthermore, intensive 1:1 interventions with the sleep psychology team results in bottlenecks in patient access. These pressures require implementation of systematised and innovative models of care, delegation to junior staff, and utilisation of time efficient (e.g., group delivery) and technological solutions designed to improve service efficiencies, effectiveness of care, and to maintain or improve patient reported experience and outcomes. A protocol for the evaluation of a new Direct to Psychology Stepped Care Model to address these pressures is presented, which utilises a direct referral pathway as well as stepped care approaches to service delivery. This study has a particular interest in exploring the feasibility and acceptability of digital intervention as a first-line treatment option.

## Methods

### Aims and objectives

The overarching aim of the current study is to evaluate the impact of adopting a new Direct to Psychology Stepped Care Model into the multidisciplinary sleep service. The model of care will incorporate a “Direct to Psychology Insomnia” referral pathway, with a “Stepped Care” treatment approach that utilises digital, group, trainee and clinician level interventions, flexibly delivered across the continuum of care. The RE-AIM Framework [20, 21] will be adopted to evaluate individual and service level outcomes in a pre- post study design comparing Standard Care to Direct to Psychology Stepped Care. The objective is to capture all service events, clinical outcomes and quality of life changes for patients referred to the sleep psychology service in order to perform a full evaluation including a cost effectiveness analysis.

The specific hypotheses include the following:

Compared to Standard Care, the Direct to Psychology Stepped Care Model will be associated with:

1. Reduced wait times and improved access to treatment
2. Equivalent (non-inferior) clinical outcomes and changes in quality of life
3. Service delivery that is less resource intensive and delivered at a lower cost

In addition, we hypothesize that stakeholders (referrer, patient, staff) will be satisfied with the service changes.

### Design and setting

**Fig. 1:**
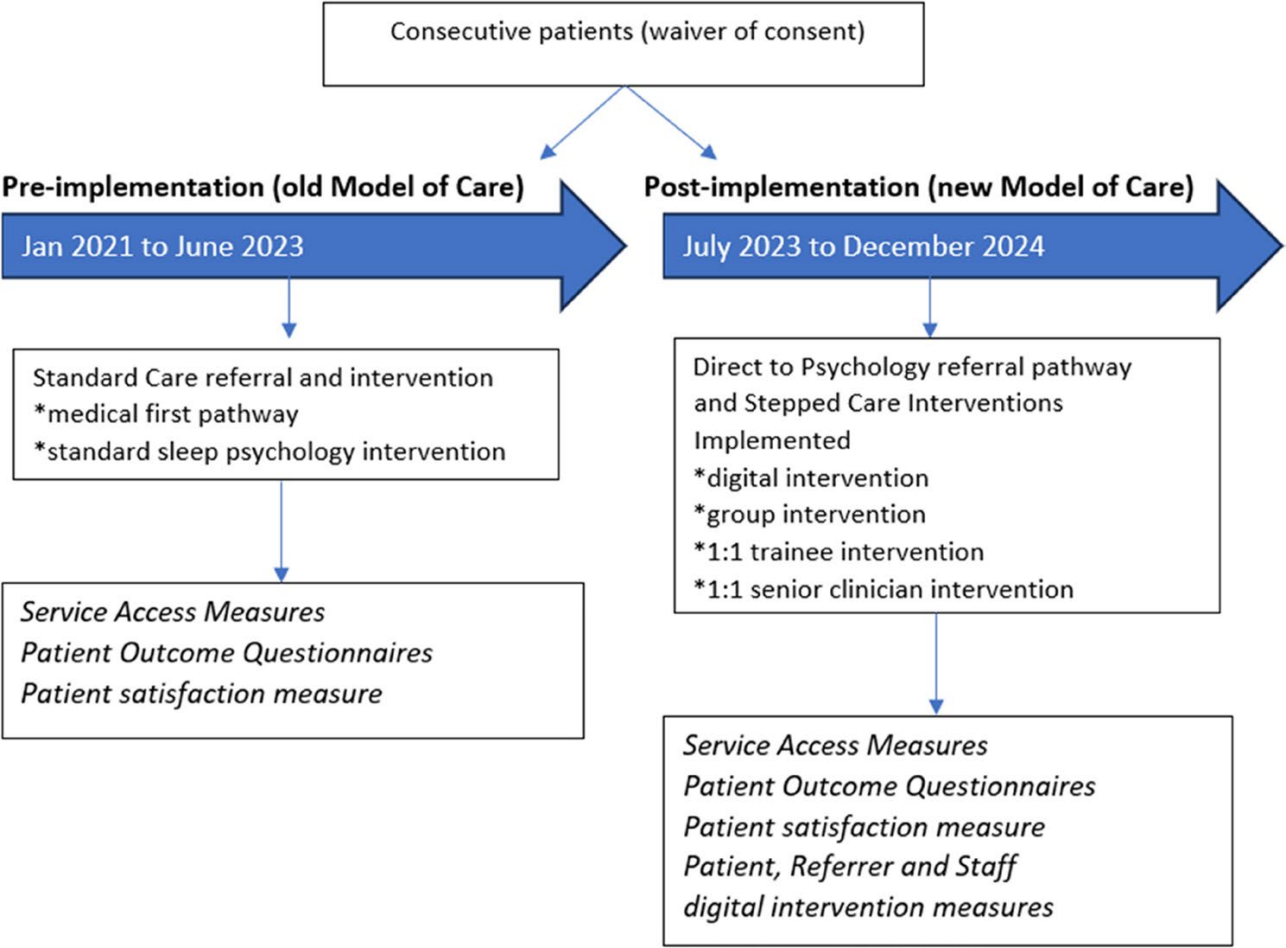
Study Flow

This project will employ a single-site, multi-phase, mixed-methods approach to evaluate the implementation of the new model of care into the Sleep Psychology service in a tertiary public hospital, embedded within the RE-AIM implementation evaluation framework [19, 20, 22]. Reporting will align with the Standards for intervention Reporting Implementation Studies (STaRI) framework [23]. Patient, service-level and implementation outcomes will be evaluated by comparing patients attending the service from 1 January 2021 until 30 June 2023 as a pre-measure (old model of care) to patients attending the service from implementation of the model from 1 July 2023 to 31 December 2024 as a post-measure (new model of care). This project has received ethics approval via the low and negligible risk pathway with a waiver of informed consent with the Darling Downs Human Research Ethics Committee (HREC 2022 QTDD 88350). Figure 1 outlines the study flow.

### Participants

Participants are patients, staff and referrers to the sleep centre. The inclusion and exclusion criteria are broad in order to maximize the generalizability and applicability of our results to the real-world health care context.

#### Patients

Consecutive patients attending the Sleep Psychology service from 1 January 2021. Inclusion criteria will include all NEW patient referrals to the Sleep Disorders Service at the Prince Charles Hospital in Brisbane, Australia who would have been referred to Sleep Psychology at some point in their patient journey from 1 January 2021 until 31 December 2024. Data collection will continue until December 2025 as patients filter through the treatment model. Exclusion criteria will include any patients referred prior to 1 January 2021 or after completion of the study.

#### Staff

Clinicians and team members, who agree to participate in our surveys.

#### Referrers

All referrers linked to patients included in the project, who respond to our surveys.

### Intervention

#### Standard care

An exploratory qualitative analysis of consumer and stakeholder perspectives to benchmark the existing service was undertaken, followed by stakeholder consensus groups that informed the new model of care parameters including inclusion and exclusion criteria for the Direct to Psychology Pathway [24], and inclusion and exclusion criteria for assigning patients to the ‘steps’ of the stepped care model [25]. In the new Model of Care, all new referrals to the Sleep Disorders Centre will be triaged by a Sleep Physician (as per current practice) and referrals will then be directed to either Standard Care and waitlisted for a Sleep Physician initial consultation, or to the “Direct to Psychology” pathway. Note that referrals to psychology will also be accepted via the Standard Care pathway for patients requiring a medical first contact and directed to the appropriate Stepped Care treatment option.

#### Direct to psychology stepped care

All patients referred to sleep psychology will complete an initial assessment according to Diagnostic and Statistical Manual (DSM-V-tr) and International Classification of Diseases (ICD-11) criteria [26, 27], including outcome measures (see *Measures).* For patients referred ‘Direct to Psychology’ additional measures will manage any potential risks associated with psychology as first contact within the model due to potential ‘missed’ medical clinical indications. These measures will include screening tools for Obstructive Sleep Apnoea (OSA) (using items from the STOP-BANG [28] and Berlin Apnoea [29] Questionnaires), hypersomnolence disorders (using items from the Cataplexy Questionnaire [30] and questions related to the presence of neuromuscular conditions, respiratory conditions and driving risk.

The inclusion criteria for Direct to Psychology criteria as defined in stakeholder co-design activities [24] include;

(i) being referred for insomnia and/or sleep psychology, and/or
(ii) suboptimal adherence to OSA treatment including continuous positive airway pressure therapy (CPAP) or mandibular enhancement splint (MAS) if medical management has been already optimised.

Exclusion criteria include;

(i) significant new/changed sleep disordered breathing (or identified significant sleep disordered breathing)
(ii) highly comorbid patients (e.g., neuromuscular conditions/weakness, respiratory conditions), diagnosis of hypersomnia/question of central disorder of hypersomnolence
(iii) category 1 (urgent) referrals
(iv) driving risk.

Patients identified as meeting the inclusion and exclusion criteria will proceed with Psychology-first intervention as indicated. Patients who meet the exclusion will be redirected back to the Standard Care model and may be re-triaged by the attending medical officer based on the additional information gleaned from the psychological assessment.

**Fig. 2:**
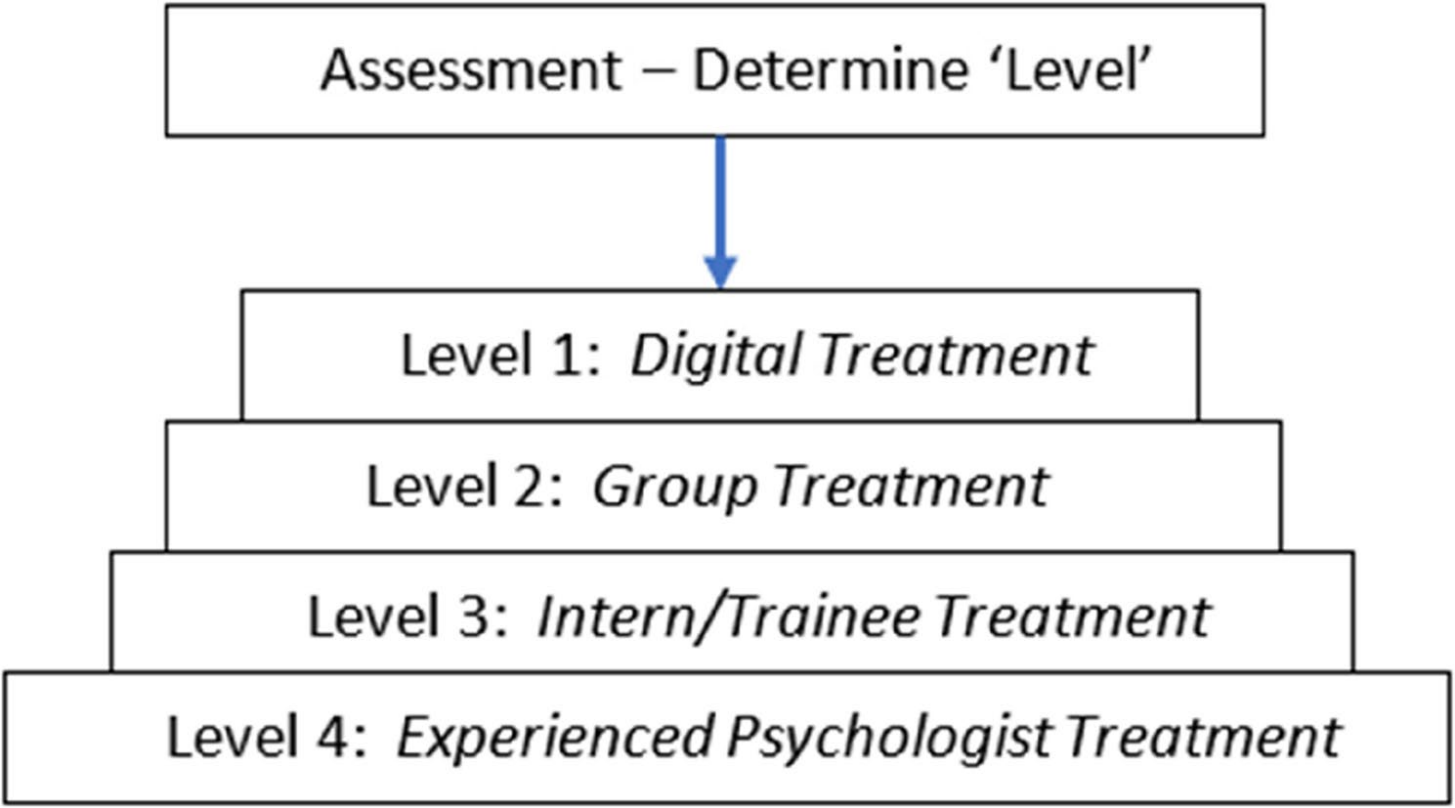
Treatment Steps

Patients progressing to psychological treatment will be matched to the appropriate treatment level. Depending on patient engagement and participation with the treatment level, as well as clinical outcomes on key assessment measures; discharge, on-referral and/or entry to another intervention level will be provided. The proposed steps of care in the new model are outlined in Fig. 2.

### Level 1: digital treatment - “Managing Insomnia"

Managing Insomnia is an evidence-based insomnia intervention, free by clinician prescription, developed by “This Way Up” - a digital therapies service through St Vincent’s Hospital, Sydney, Australia [17]. This therapy consists of 4 sessions, which the client guides themselves through at their own pace. Each session takes about one hour to complete and is designed to be completed at one session per week. Participants have up to 90 days to complete the program once they start. The sessions are presented in a comic-book like format, and patients are prompted to complete questionnaires and sleep diaries throughout the sessions to facilitate engagement with the material. The referring Psychologist is able to track the progress of the patient, including their commencement of the program and completion of each session, as well as their outcome measures collected at each session (Insomnia Severity Index and K-10 score). A brief check-in phone call 2 weeks after treatment prescription will be undertaken to check progress and support engagement with the program as needed. No therapeutic content will be delivered during the phone call [31]. If the patient is engaging well with the intervention, a subsequent phone call will be scheduled for another six weeks to coincide with treatment conclusion and to collect outcome measures. If the patient has not commenced the intervention at the initial 2 week check-in, or if the patient reports barriers to engagement, a second check-in phone call will be scheduled 2 weeks later. If the patient has not engaged with the digital therapy within 4 weeks of being prescribed the intervention, reasons for this will be discussed and options for discharge, on-referral and/or entry to another intervention level will be provided.

### Level 2: group program

The manualised CBTi intervention and sleep manual delivered during group intervention was refined by our Sleep Psychology team from an evidence-based insomnia treatment developed at Flinders University, Adelaide, Australia [32]. The intervention is 4 sessions and covers the key components of insomnia treatment – psychoeducation, sleep restriction therapy, stimulus control, cognitive therapy and relaxation training. Each session is 90 min, and there are a maximum of 6 participants in each group session. The experienced Psychologist and Intern Psychologist monitor participation and clinical outcomes during the group program.

### Level 3: 1:1 with sleep psychology intern

Patients assigned to this level will undergo treatment 1:1 with the Psychology Intern under supervision from the experienced Psychologist. Psychology interns/trainees have completed undergraduate psychology training and are post-graduate (Masters, Doctorate or PhD) students on clinical placement. The CBTi intervention described above for Level 2 may be provided along with additional evidence-based strategies as required depending on sleep or mental health comorbidities; such as cognitive behavioural therapy (CBT) for depression/anxiety, dream rescripting for nightmares, and Motivational Interviewing for CPAP adherence [33] for those with comorbid OSA. Each of these sessions are 60 min in duration and delivered at a weekly to fortnightly interval. The number of sessions depends on how many sessions the patient requires to achieve adequate symptom relief based on ongoing assessment and monitoring. Participation and clinical outcomes are monitored throughout.

### Level 4: 1:1 with experienced Psychologist

Patients assigned to this level will undergo treatment 1:1 with the experienced Psychologist. The experienced Psychologist will monitor participation and clinical outcomes during the 1:1 intervention program, and provide additional evidence -based strategies depending on sleep or mental health comorbidities (as described in Level 3 above). Discharge or on-referral will be provided at the conclusion of the treatment.

For Levels 2 to 4 - sessions will be delivered flexibly either face-to-face or via telehealth.

### Measures

Primary and secondary outcomes will be collected at two endpoints; pre- and post- service change, comparing the pre-implementation period as a pre-measure (old model of care) to those participating in the (NEW) model of care as the post-measure.

#### Primary outcomes

##### Service access measures

Access measures include new patient activity for the sleep psychology service, and time from referral to treatment commencement and discharge within the sleep psychology service when comparing patients treated under the old model of care compared to those treated under the new model of care. Number of clinical occasions of service will be collected for the sleep psychology service and the medical service and compared for those attending under the old model of care compared to those attending under the new model of care. 

##### Patient-related outcomes

Patient-related primary outcomes will test non-inferiority in change scores on key clinical outcome measures from pre- to post- intervention when comparing those patients treated under the old model care compared to those treated under the new model of care. Furthermore, % patients treated adequately at each step of the Stepped Care Model will be measured by the number of patients discharged from the service by the treating clinician with outcome measures indicating reliable clinical change from pre- to post- intervention. The Clinical outcome measures include the Insomnia Severity Index (ISI) [34], Depression Anxiety and Stress Scale (DASS-21) [35], Kessler Psychological Distress Scale (K10) [36], and the EuroQol (EQ-5D-5 L) [37].

**Insomnia Severity Index** (ISI) is a 7-item self-report questionnaire assessing the nature, severity, and impact of insomnia over the past two weeks on a 5-point scale from 0 = no problem; 4 = very severe problem. The measure is widely used in the literature as well as clinically where a score of 11 is indicative of likely meeting criteria for an insomnia disorder in clinical trials and a change score greater than 8 is the minimally important difference to be considered ‘markedly improved’ or in remission [34, 38].

**Depression Anxiety and Stress Scale** (DASS-21) is a short version of a 42-item self-report instrument designed to measure three related negative emotional states: depression, anxiety and tension/stress over the past 7 days. Items are scored on a 3-point scale from 0 (did not apply to me at all) to 3 (applied to me very much, or most of the time). Total scores represent symptoms of distress in each of the domains in the ‘normal’, ‘mild’, ‘moderate’, ‘severe’ and ‘extremely severe’ relative to population norms [35].

**Kessler Psychological Distress Scale** (K10): The K10 is a 10-item self-report scale to assess symptoms over the past 4 weeks. Items are rated on a 5-point scale from 1 (none of the time) to 5 (all of the time). A score of less than 20 represents low psychological distress ‘likely to be mentally well’. Between 20 and 24 represents moderate distress ‘may have a mild mental disorder’ and between 25 and 29 represents high psychological distress ‘likely to have a moderate mental disorder’ [36].

**EuroQol** (EQ-5D-5 L): The EQ-5D-5 L measures five dimensions of quality of life: mobility, self-care, usual activities, pain/discomfort and anxiety/depression. Each dimension has 5 levels: no problems, slight problems, moderate problems, severe problems, and extreme problems. The EQ VAS records the patient’s self-rated health on a vertical visual analogue scale where the endpoints are labelled ‘The best health you can image (100)’ and ‘The worst health you can image (0)’. The EuroQol also allows preliminary health economics evaluation by performing quality-adjusted life year calculations as a measure of cost-effectiveness of the intervention compared to standard care [37].

### Secondary outcomes

#### Patient satisfaction

To test for non-inferiority in satisfaction with the provided service measured when comparing those patients treated under the old model care compared to those treated under the new model of care, a feedback informed treatment tool will be utilised.

##### Session Rating Scale (SRS)

The Session Rating Scale (SRS) is four-item visual analogue scale with each scale measured between 0 and 10 designed to assess key dimensions of effective therapeutic relationships and global satisfaction with treatment. The SRS is scored by summing score on each of the four scales with a total possible score of 40 [39].

#### Patient, staff and referrer satisfaction

We are particularly interested in incorporating digital intervention into our model of care. Therefore, for those patients assigned to digital intervention in the new model of care, acceptability and relatability of the online program will be measured via post-intervention questionnaires delivered to patients and staff via a RedCap survey. All multidisciplinary staff of the Sleep Disorders Centre employed during the new model of care implementation phase will be send a questionnaire via Microsoft Forms. Note that these questionnaires are modelled on the service evaluation questionnaires from the “Reboot” online pain management program implemented at Queensland Health’s Sunshine Coast Persistent Pain Management Service [31].

### Implementation and evaluation – RE-AIM

To measure the impact of our service change on both patient and service/organisational level outcomes, the evaluation is outlined below with according to the RE-AIM framework (see Table 1).

**Table 1:** RE-AIM evaluation framework

Evaluation Question	RE-AIM Domain	Indicator/Measure	Standard/Target	Data Source
*Did patients access care as expected under the new model of care?*	**Reach – The absolute number**,** proportion**,** and representativeness of individuals who are willing** **to participate**	• Percentage of patients agreeing to digital compared to those offered • Patient-reported reasons for declining digital intervention • Percentage of patients referred Direct to Psychology who remain on this pathway • Differences in patient characteristics (age, sex, geographical location, presenting issue) between those agreeing to digital compared to those declining • Number of patients and patient characteristics (age, sex, geographical location, presenting issue) of those assigned to each treatment level • Differences in patient characteristics (age, sex, geographical location, presenting issue) between those referred Direct to Psychology compared to those referred via the standard pathway • Time to start the online program from date of prescription for those assigned to digital intervention • % digital intervention sessions completed for those assigned to digital intervention	> 80% patients offered digital intervention accept digital < 20% patients are referred back to standard care > 80% patients commence within 4 weeks > 80% patients complete digital intervention	Patient qualitative and quantitative surveys Medical Record Patient registration system
*How effective was the model of care in achieving its intended outcomes?*	**Effectiveness** – impact on outcomes and quality of life/economic outcomes	**Primary outcomes** *Service Access Measures* • Average number of occasions of Service within Sleep Psychology service under ‘old’ model compared to ‘new’ model of care • Percentage of new patient activity under ‘old’ model compared to ‘new’ model of care • Average wait-time to access a first appointment with Sleep Psychology from date of referral under ‘old’ model compared to ‘new’ model of care • Average time to discharge from first appointment to final appointment with Sleep Psychology under ‘old’ model compared to ‘new’ model of care • Percentage of patients discharged from psychology *Patient-related Outcomes* • Change scores on clinical outcome measures pre- to post- treatment on clinical outcome measures (ISI, DASS-21, K10 and EQ-5D-5 L) comparing patients treated under ‘old’ model versus ‘new’ model of care • % patients treated adequately at each level of Stepped Care • Differences in patient characteristics (age, sex, geographical location, presenting issue) between those who are adequately treated at each level of the new model of care (moderation analysis) *Economic Outcomes* • Quality-adjusted life year calculations as a measure of cost-effectiveness of the ‘new’ model of care compared to the ‘old’ model of standard care **Secondary outcomes** *Patient satisfaction* • Patient satisfaction measure (SRS) post- treatment comparing patients treated under ‘old’ model versus ‘new’ model of care *Patient satisfaction with digital intervention* • Patient reported ease of access to digital intervention • Acceptability and relatability of the online program/service provided (rated by patients), including: i. motivation to access online treatment (diversion from public hospital services for insomnia treatment) ii. understanding of intervention materials iv. perceived change in sleep problem • Differences in patient characteristics (age, sex, geographical location, presenting issue) between those who complete the digital intervention compared to those who do not complete	Significantly fewer occasions of service under new model of care Significantly greater % new patient appointments under new model Significantly shorter wait-time to first appointment under new model Significantly shorter time to discharge under new model Outcomes are non-inferior under new model of care; upper bound 95%CI on outcome measures is less than MCID MCID is achieved on outcome measures by > 80% patients at each level The ‘new’ model is more cost effective Patient reported satisfaction is non-inferior under new model of care; upper bound 95%CI is less than MCID > 90% patients report positive experiences	Patient qualitative and quantitative surveys Medical Record Patient registration system
*How effective was the model of care in increasing stakeholder engagement in Direct to Psychology (for staff) and digital intervention referrals (for staff and referrers)?*	**Adoption – engagement with and willingness to implement the new model of care**	• Percentage of patients referred to Psychology who were eligible for Direct to Psychology are referred to this pathway **Secondary outcomes** *Staff and Referrer satisfaction with digital intervention* • Staff and referrer reported ease of access to digital intervention • Acceptability and relatability of the online program/service provided including: i. motivation to refer patients to online treatment (diversion from public hospital services for insomnia treatment) ii. understanding of intervention materials iii. percentage of referrers and staff who would engage with online treatments in their standard care iv. perceived change in understanding of insomnia management	> 80% patient meeting inc/excl criteria are referred appropriately > 90% staff and referrers report positive experiences > 90% staff and referrers report they are ‘somewhat motivated’, ‘very motivated’ or ‘extremely motivated’ to prescribe the digital intervention in their practice	Staff and referrer qualitative and quantitative surveys Medical Record Patient registration system
*Was the model was delivered as intended?*	**Implementation -**consistency and fidelity of delivery as intended and the time and cost of the intervention	• % of model of care elements delivered as per protocol • Differences in % of model of care elements delivered as per protocol compared between participating intervention staff • Differences in patient-related outcomes (%MCID achieved) compared between participating intervention staff • Number of psychology-led discharges from Direct to Psychology pathway (diversion from medical pathway) • Number of occasions of service per psychology resource (staffing) available comparing the ‘old’ and ‘new’ model of care • Direct service costs versus revenue generated for new and review consultations of the ‘new’ model of care compared to the ‘old’ model of standard care	> 90% intervention components are delivered as per the protocol Implementation adaptations are reported at dissemination No significant difference in % model of care elements delivered between intervention staff No significant difference in MCID achieved between intervention staff The new model of care is cost effective, less resource intensive and more time efficient than the old model of care	Medical Record Patient registration system Document review
*How sustainable is this model of care*,* how well can the model of care continue to operate into the future?*	**Maintenance – long term effects of the program**,** model is business as usual**	• % components of model still operating 6 months post completion of the study • Description of which components of the model are still operating 6 months post completion of the study • Demand for the service – number of Direct to Psychology referrals and patient assignment to the different ‘levels’ of the model 6 months post completion of the study • Evidence of scale and spread of similar models across other services internal and external to the health provider	> 95% of the model is still operating 6 months post completion (July 2026) Number of Direct to Psychology referrals and patient assignment to treatment levels are sufficient to justify ongoing service provision	Document review

### Statistical analysis

The project will employ a mixed methods approach. Qualitative surveys for patients, multidisciplinary staff, and referrers was undertaken pre-service change to ascertain the acceptability of the digital therapy, as well as familiarity, access, confidence, and relatability of this treatment option [25]. Post-implementation surveys will also be undertaken on these same metrics. Quantitative data will be collected pre- and post-service change to ascertain treatment effectiveness in terms of clinical outcome measures, as well as service measures of interest such as patient flow, occasions of service and program engagement. Based on similar studies [40], and allowing for some incomplete data, we expect to have data for approximately 110 participants from each pre- and post- time period, resulting in a sample of 220 participants overall who will be suitable for the Direct to Psychology pathway. To assess wait times, participants will be categorised into four groups based on the time-period (pre/post implementation) and whether they met eligibility criteria for the Direct to Psychology pathway. Differences in wait times between the groups will be assessed using a one-way Analysis of Variance (ANOVA) and linear regression modelling. In a one-way ANOVA study, a sample of 220 participants, divided by 4 groups, achieves 80% power with a significance level of 0.05 to detect a moderate-large effect size (Cohen’s f) of 0.25 [41]. The effects of other variables of interest and potential confounders (patient characteristics, diagnosis,) will be tested in multivariable models. For differences in patient outcome measures pre- and post- intervention when comparing pre- and post- service change, the mean between-group difference and 95% confidence interval (CI) will be calculated. As this aspect of the evaluation is a non-inferiority trial (that is, we expect clinical outcomes to not be reduced due to the service change), the criteria adopted to determine non-inferiority will be if the upper bound 95%CI on each clinical outcome measure was less than the minimal clinical important difference (MCID). Quantitative data measuring pre- and post- service change outcomes on key indices of patient service satisfaction, service characteristics (e.g., occasions of service); as well as referrer and staff service satisfaction, and engagement with digital therapies, will be evaluated via univariate statistical tests where indicated such as independent samples t-test (or nonparametric equivalent), and categorical comparisons will be evaluated using Pearson’s chi-square. The distribution of outcome measures will be assessed, and appropriate transformations will be applied for use in modelling. Differences between group means will be tested using generalised linear regression modelling. Health related quality of life will be measured pre- and post- implementation using the EQ-5D-5 L. Population norms for EQ-5D-5 L Australia will be used to value the data set [42].

### Data management

All data collected throughout the project will be de-identified at the point of extraction. A unique study and participant ID code will be assigned to each record. The linkage file connecting identifiers to participant information will be held securely by the Principal Investigator (SW).

Data entry and coding procedures will follow standardised protocols. Range checks and validation rules will be applied to data fields to prevent entry errors and maintain data consistency.

All digital data will be stored on encrypted, password-protected servers compliant with institutional data security policies. Access to the data will be limited to authorised personnel only. Physical files will be stored in locked filing cabinets within secure, access-controlled research offices.

Further detail regarding data handling procedures—including the data management plan, coding framework, and quality assurance processes—is available from the study team upon reasonable request and is maintained in the internal project documentation, separate from the protocol.

The scope of the study does not warrant the additional oversight of a Data Monitoring Committee (DMC). Instead, data oversight and integrity monitoring will be managed internally by the study team under the supervision of the PI, in line with institutional governance procedures.

#### Dissemination policy

There are no publication restrictions imposed by the sponsor or any funding body. The investigators retain the right to publish and disseminate findings regardless of the outcomes.

Authorship will be determined based on the NMHRC authorship guidelines. Professional medical writers will not be used in the development of the final publication. Participant-level de-identified datasets and statistical code may be made available to qualified researchers on reasonable request, subject to ethical approvals and data sharing agreements that ensure participant confidentiality and appropriate data use.

## Discussion

The overarching goal of this project is to implement and report on changes in the service model in our multidisciplinary sleep service, specifically a new model of care that incorporates a “Direct to Psychology Insomnia” referral pathway, that includes a “Stepped Care” treatment approach that utilises digital, group therapy, and intern and senior therapist-led interventions. Other multidisciplinary teams have envisioned similar Allied Health-led referral pathway plans to beneficial effect [43, 44]. Results from this study will highlight the potential for Direct to Psychology referral pathways in reducing the clinical burden on physicians and expediting patient access to first-line treatment for insomnia. Patient outcomes, satisfaction, and service-based evaluation tools are embedded in this project to compare the model of care to treatment as usual. We anticipate that if shown to be acceptable, the findings from this study will inform future iterations and enhancements to the model of care, including the scalability to apply the model to other services.

Streamlining sleep psychology interventions into a stepped care treatment approach will allow patients to access the appropriate level of care based on their level of acuity and clinical presentation, with the model being flexible enough for patients to be expedited to more resource intensive levels of care based on treatment response. The new model of care will utilise technology as part of a Stepped Care model to provide a platform for more efficient service delivery, backed by expert clinician oversight in order to provide care to more patients within existing resources. We expect the new evidence-based model of care will substantially reduce wait times for patients and provide timely access to effective Psychological treatment, resulting in non-inferior clinical outcomes. We posit that this dynamic approach to sleep disorder management will result in better patient access and outcomes, leading ultimately to reduced societal burden from reduced productivity and health care costs due to untreated sleep disorders.

By incorporating consumer and stakeholder engagement throughout, we will ensure the model of care developed is acceptable to all stakeholders. By sharing and disseminating the learnings from this program to primary health services and the general community, we aim to increase engagement in evidence-based digital therapies, thus increasing the baseline sleep health of our community and reducing reliance on acute hospital services. By increasing access to, and promoting the availability of evidence-based digital therapies, we can empower consumers to engage with their own self-management, potentially reducing referrals into the acute hospital system.

Given the real-world dataset and service imperatives to be utilised, there are potential limitations to this study, including challenges in recruiting evenly to all intervention arms, and unplanned but typical participant attrition during the self-guided portion of the digital intervention, both of which may impact the results. We plan to address these challenges for those assigned to digital intervention via bi-monthly therapist or intern ‘check-in calls’ to address barriers to engagement, and to make administrative changes to triage and intake processes to flow more participants to the digital arm if indicated. However, the study is adequately powered to compensate for these factors and we anticipate that the Direct to Psychology referral pathway and Stepped Care treatment model will be acceptable to patients and clinicians and overall will improve patient care within the health system.

## Supplementary Information

Supplementary Material 1.

## Data Availability

Data and materials that support this manuscript are available from the corresponding author, SW, upon reasonable request.

## Abbreviations

AASM: American Academy of Sleep Medicine
ACTRN: Australian and New Zealand Clinical Trials Registry
ASA: Australasian Sleep Association
CBT: cognitive behavioural therapy
CBTi: Cognitive Behaviour Therapy for Insomnia
COMISA: Comorbid Insomnia and Obstructive Sleep Apnoea
CI: Confidence Interval
CPAP: Continuous Positive Airway Pressure therapy
DASS-21: Depression Anxiety and Stress Scale
DSM-V-tr: Diagnostic and Statistical Manual
EQ-5D-5L: EuroQol
HREC: Human Research Ethics Committee
ISI: Insomnia Severity Index
ICD-11: International Classification of Diseases
K10: Kessler Psychological Distress Scale
MAS: Mandibular Enhancement Splint
MCID: minimal clinical important difference
MoC: Model of Care
OSA: Obstructive Sleep Apnoea
SRS: Session Rating Scale
StaRI: Standards for intervention Reporting Implementation Studies
